# A multi gene-approach genotyping method identifies 24 genetic clusters within the genotype II-European African swine fever viruses circulating from 2007 to 2022

**DOI:** 10.3389/fvets.2023.1112850

**Published:** 2023-01-25

**Authors:** Carmina Gallardo, Nadia Casado, Alejandro Soler, Igor Djadjovski, Laura Krivko, Encarnación Madueño, Raquel Nieto, Covadonga Perez, Alicia Simon, Emiliya Ivanova, Daniel Donescu, Vesna Milicevik, Eleni Chondrokouki, Imbi Nurmoja, Maciej Frant, Francesco Feliziani, Petr Václavek, Simona Pileviciene, Arias Marisa

**Affiliations:** ^1^European Union Reference Laboratory for ASF (EURL-ASF): Centro De investigación en Sanidad Animal (CISA-INIA, CSIC), Madrid, Spain; ^2^Faculty of Veterinary Medicine, University Ss. Cyril and Methodius in Skopje, Skopje, North Macedonia; ^3^Latvia NRL: Laboratory of Microbiology and Pathology, Institute of Food Safety, Animal Health and Enviroment, BIOR, Riga, Latvia; ^4^Bulgaria NRL: National Diagnostic and Research Veterinary Medical Institute (NDVRI), Sofia, Bulgaria; ^5^Romania NRL: Institute for Diagnostic and Animal Health, Bucharest, Romania; ^6^Republic of Serbia NRL: Institute of Veterinary Medicine of Serbia, Belgrade, Serbia; ^7^Greece NRL: Greek Ministry of Rural Development and Food FMD, Virological, Rickettsial & Exotic Diseases, Athens, Greece; ^8^Estonian NRL: National Centre for Laboratory Research and Risk Assessment (LABRIS), Tartu, Estonia; ^9^Poland NRL: National Veterinary Research Institute, Puławy, Poland; ^10^Italy NRL: Istituto Zooprofilattico Sperimentale (IZS) dell'Umbria e delle Marche, Perugia, Italy; ^11^Czech Republic NRL: State Veterinary Institute Jihlava, Jihlava, Czechia; ^12^Lithuania NRL: National Food and Veterinary Risk Assessment Institute (NFVRAI), Vilnius, Lithuania

**Keywords:** ASFV, genotyping, TRS, SNP, genetic groups

## Abstract

**Introduction:**

African swine fever (ASF) is a contagious viral disease of pigs and wild boar that poses a major threat to the global swine industry. The genotype II African swine fever virus (ASFV) entered the European Union (EU) in 2014 and since then fourteen countries have been affected, Italy and North Macedonia being the last in 2022. While whole genome sequencing remains the gold standard for the identification of new genetic markers, sequencing of multiple loci with significant variations could be used as a rapid and cost-effective alternative to track outbreaks and study disease evolution in endemic areas.

**Materials and methods:**

To further our understanding of the epidemiology and spread of ASFV in Europe, 382 isolates collected during 2007 to 2022 were sequenced. The study was initially performed by sequencing the central variable region (CVR), the intergenic region (IGR) between the *I73R* and *I329L* genes and the *O174L* and *K145R* genes. For further discrimination, two new PCRs were designed to amplify the IGR between the *9R* and *10R* genes of the multigene family 505 (MGF505) and the IGR between the *I329L* and *I215L* genes. The sequences obtained were compared with genotype II isolates from Europe and Asia.

**Results:**

The combination of the results obtained by sequencing these variable regions allowed to differentiate the European II-ASFV genotypes into 24 different groups. In addition, the SNP identified in the IGR *I329L*-*I215L* region, not previously described, grouped the viruses from North Macedonia that caused the 2022 outbreaks with viruses from Romania, Bulgaria, Serbia and Greece, differentiating from other genotype II isolates present in Europe and Asia. Furthermore, tandem repeat sequence (TRS) within the *9R*-*10R* genes of the multigene family 505 (MGF505) revealed eight different variants circulating.

**Discussion:**

These findings describe a new multi-gene approach sequencing method that can be used in routine genotyping to determine the origin of new introductions in ASF-free areas and track infection dynamics in endemic areas.

## 1. Introduction

African swine fever (ASF) is considered one of the most devastating disease of pigs and wild boar. The ASF virus (ASFV) is a large, enveloped virus, member of the family Asfarviridae (1). The genome of ASFV is a linear double-stranded DNA (dsDNA) molecule with a length of 171–193 kb with terminal inverted repeats and hairpin loops (2). The size differences between the different strains are due to insertions or deletions at the terminal regions of the genome where the multigene families (MGF) are located. Variations in the conserved central region related to single nucleotide polymorphism (SNP) or the presence of tandem repeat sequences (TRS) have also been described (2). ASFV isolates are classified into 24 genotypes by comparative analysis of the C-terminal end of the *B646L* gene, which encodes the p72 protein (3, 4). All 24 genotypes are present in Africa, where ASF was first described a century ago (5). Outside of Africa, genotype I was related to historical ASFVs circulating in Europe and America until the mid-1990s. This genotype has remained endemic only in Sardinia (Italy) since 1978, although its presence has been recently associated to domestic pig's outbreaks in China in 2021 (6).

In 2007, the presence of ASFV genotype II was confirmed in the Caucasus region of Georgia (7). From there, ASFV gradually spread to neighboring countries (i.e., Armenia, Azerbaijan, Russia, Ukraine, Moldova, and Belarus) affecting domestic pigs and wild boar. In the European Union (EU) the presence of ASFV genotype II was first reported in 2014 in Lithuania and Poland (8). Since then, genotype II of ASFV has been notified in Belgium, Bulgaria, the Czech Republic, Estonia, Germany, Greece, Hungary, Italy, Latvia, Lithuania, Poland, Romania, and Slovakia, causing serious concerns. So far, only two European countries have managed to eradicate the disease: Belgium (event resolved in March 2020) and the Czech Republic (event resolved in April 2018). Furthermore, no ASF outbreaks in domestic pigs nor cases in wild boar have been reported in Greece since February 2020. The disease has also been reported in Serbia and North Macedonia, so there is a constant risk of re-introduction for European countries that are sharing borders (9). In August 2018, ASFV genotype II was detected in China (People's Republic of), marking the first occurrence of ASF in Asia (10). As of the end of October 2022, ASF has been reported in 32 provinces in China and 16 Asian countries; the last Thailand in January 2022. In September 2019, the ASFV appears in Oceania, in Timor-Leste, followed by Papua New Guinea (March 2020). In July 2021, another transcontinental leap in ASF occurs with the reappearance of genotype II in the Americas after an absence of almost 40 years, with outbreaks detected in the Dominican Republic and Haiti (11).

The key for understanding the diversity of the ASFV, including its evolution, is to analyze its genetic variations by sequencing specific genetic markers. The carboxy terminal end of the p72 gene (*B646L*) is sequenced to place ASFVs within one of 24 genotypes (3, 4, 12). This method allows relatively quick and easy typing of ASFV strains and remains the first method to identify the origin of an outbreak in case of introduction into new territories. However, the *B646L* gene-based genotyping method does not always provide adequate typing resolution or the ability to discriminate between closely related viruses. For intra-genotypic differentiation, the central variable region (CVR) of the *B602L* gene, is one of the most widely used markers. It is characterized by the presence of tandemly repeated sequences (TRS) which allows up to 31 subgroups of ASFV to be distinguished (13). However, despite the large number of ASFV genotype II outbreaks in the EU, only three CVR variants have been identified in Estonia, with variant 1 (Georgia 2007-type) being predominant throughout the EU (14). The analysis of additional genetic markers such as TRS present in the *O174L* gene (15, 16), or in the intergenic regions (IGR) between *I73R*-*I329L* (8), has made possible to differentiate between closely related genotype II viruses (17–19). Similarly, sequencing of single nucleotide polymorphisms (SNPs) within the *K145R* gene has also been described as a useful molecular tool to track the spread of ASFV in Poland (16).

It was hypothesized that these regions could be used as a rapid and cost-effective method to investigate the epidemiology, evolution, and molecular relationship of a large number of EU strains. The aim of this study was therefore to open the spectrum of characterized viruses and perform a molecular genotyping of 382 ASFV isolates from Europe, collected during 2007 to 2022. The study was performed with the initial analysis of the TRS located in the CVR, in the IGR between *I73R*–*I329L* and in the *O174L* gene, and by the sequencing of the SNP identified in the *K145R* gene. For additional discrimination, two new PCRs were designed to amplify new markers characterized by the presence of TRS, the IGR between the *9R*−*10R* genes of the multigene family 505 (MGF505) (20, 21), and the IGR between the *I329L* and *I215L* genes (22).

This is the first detailed report on the molecular characterization of the ASFV strains circulating in all affected EU countries since 2014 to 2022. Moreover, the multi-gene approach strategy followed in this study distinguish 24 genetic groups and revealed previously undescribed variants circulating in the EU that allowed us, for the first time, to trace variants with genomic epidemiology to regional clusters.

## 2. Materials and methods

### 2.1. Samples selection

A total of 345 clinical samples collected in the EU from ASFV-positive wild boar (*n* = 244) and domestic pigs (*n* = 101) between 2014 and 2022 sent by the EU National Reference Laboratories (NRLs) to the EU Reference laboratory (EURL) for ASF (CISA, INIA-CSIC, Madrid Spain) for ASF laboratory confirmation were included in this study. Specimens were selected to represent all geographic areas from affected EU countries, including index cases, where ASFV has been present up to now. Thirty seven ASFVs from the neighboring non-EU Serbia, North Macedonia, Moldova, Ukraine, Belarus, Russia Federation, Armenia and Georgia were also included resulting in a final panel of 382 ASFVs characterized. The detailed characteristics of ASFVs, as well as obtained results for individual viruses, are presented in Supplementary Table S1.

### 2.2. PCR amplification for routine ASF diagnostic

To confirm the presence of the ASFV genome in samples received, the DNA was extracted from clinical samples (sera, whole blood, tissues), using the High Pure PCR Template Preparation Kit (Roche Diagnostics GmbH, Roche Applied Science, Mannheim, Germany). Briefly, 10% (w/v) clarified homogenized tissue suspensions or blood were prepared in phosphate-buffered saline (PBS). For ASF routine diagnosis, the Universal Probe Library (UPL) real-time PCR (23, 24) was performed using undiluted extracted DNA from each sample. Extracted DNA was stored at −20°C until further analysis.

### 2.3. PCR amplification for sequencing

For genetic characterization, PCR was performed on nucleic acid extracted from ASFV positive samples using published primers and protocols to initially amplify four independent regions on ASFV genome; (i) the CVR of the *B602L* gene using the CVR1 and CVR2 primer pair (26), (ii) TRS located between the *I73R* and *I329L* (IGR) genes using primers ECO1A and ECO1B (8), (iii) TRS located in the *O174L* gene using the *O174L*F and *O174L*F (15, 16), and (iv) partial *K145R* gene using primers *K145R*-F and *K145R*-R (16). Amplification conditions were previously described (8, 15, 17, 26).

We designed two additional sets of primers to amplify; (i) a 551 base pair (bp) amplicon that includes the TRS located in the IGR between the MGF505 *9R* and *10R* genes (**Figure 3A**) using primers MGF505U and MGF505L (20), and (ii) a 604 bp fragment containing the 55 bp at the 3′ end of the *I329L*, 288 bp of the IGR between the *I329L* and *I215L* genes, and 261 bp at the 5′ end of the *I215L* gene using the primers named ECO2A and ECO2B. The primer binding sites were based on the Georgia ASFV genome (Accession No. FR682468.2). Conditions for the PCR assays were as follows; 10–50 ng of sample DNA, 1x PCR buffer II (50 mM KCl, 10 mM Tris-HCl), 2.5 mM MgCl_2_, 0.2 mM concentrations of the four deoxynucleoside triphosphates (Roche Molecular Biochemicals), 0.4 μM concentrations of the primers and 0.025 U/μl of Taq Gold polymerase (Applied Biosystems). The amplification programs were identical to that used for the CVR amplification (25) but with annealing temperatures of 56°C for the IGR MGF505 *9R*-*10R* and 55°C for the IGR *I329L*-*I215L* amplification. Primers specific to selected regions were designed by Primer3 (http://primer3.ut.ee). Detailed characteristics of the primers used and regions amplified are summarized in Table 1.

**Table 1:** Characteristics of the primers used in the study.

**ID**	**Name**	**Sequence 5^′^ → 3^′^**	**Position referring to Georgia 2007/1 (FR682468.2)**	**Amplicon length (nt)**	**Reference**
CVR	CVR1	ACTTTGAAACAGGAAACWAATGATG	102,943–102,968	491	(25)
	CVR2	ATATTTTGTAATATGTGGGCTGCTG	102,520–102,524		
IGR	Eco1A	CTATTTATCCCCCRCTTTGG	173,272–173292	356	(8)
	Eco1B	TCGTCATCCTGAGACAGCAG	173,607–173,627		
*O174L*	*O174L*-F	TGGCTCAGACGATATTTCAACTC	128,160–128,182	63	(15)
	*O174L*-R	GCCTCCACCACTTGAACCAT	128,832–128,813		
*K145R*	*K145R*-F	TTTCAGGCTGAAAACTTTTTAT	65,030–65,051	22	(16)
	*K145R*-R	AAAGTTTTCAATGGTTGTTAGC	65,312–65,291		
MGF	505U	AGAAACCGCAGATGAATGTA	45,069–45,089	51	This study
	505L	TACAGCCCTAGTTGTTGAAG	45,567–45,587		
ECO2	Eco2A	TCCTACCTGTTAAGCCACTTCC	174,452–174,472	604	This study
	Eco2B	GCAAATGTGGATGCAGCTAA	175,035–175,055		

### 2.4. Sequence analysis

Amplicons of predicted size were excised and purified by Quiaex gel extraction (QUIAGEN) and the nucleotide sequence of purified products determined using the same primers as used for amplification on an automated 3730 DNA analyzer (Applied Biosystems).

Sequence quality assessment was done using Chromas (www.technelysium.com.au). The individual forward and reverse sequences were assembled using the CLUSTALW algorithm implemented in MEGA v11 software (26). The generated nucleotide sequences were compared to publicly available sequences using the Basic Local Alignment Search Tool (https://blast.ncbi.nlm.nih.gov/Blast.cgi). Multiple sequence alignment was done using the CLUSTALW algorithm implemented in MEGA v11 software. For the TRS analyses nucleotide sequence or deduced amino acid sequences were manually aligned with gaps being inserted to optimize the alignment. Comparisons were made using previous TRS as described (8, 13–16). The sequences were compared with ASFV genotype II homologous sequences from Europe and Asia available in the GenBank, giving final data sets of 540 sequences for CVR, 910 for IGR, 636 for *O174L*, 540 for *K145R*, and 442 for the MGF and ECO2 regions.

Sequences generated within this study were deposited in GenBank (Supplementary Table S1).

## 3. Results

### 3.1. Analysis of the CVR within the *B602L* gene (CVR region)

**Figure 1:**
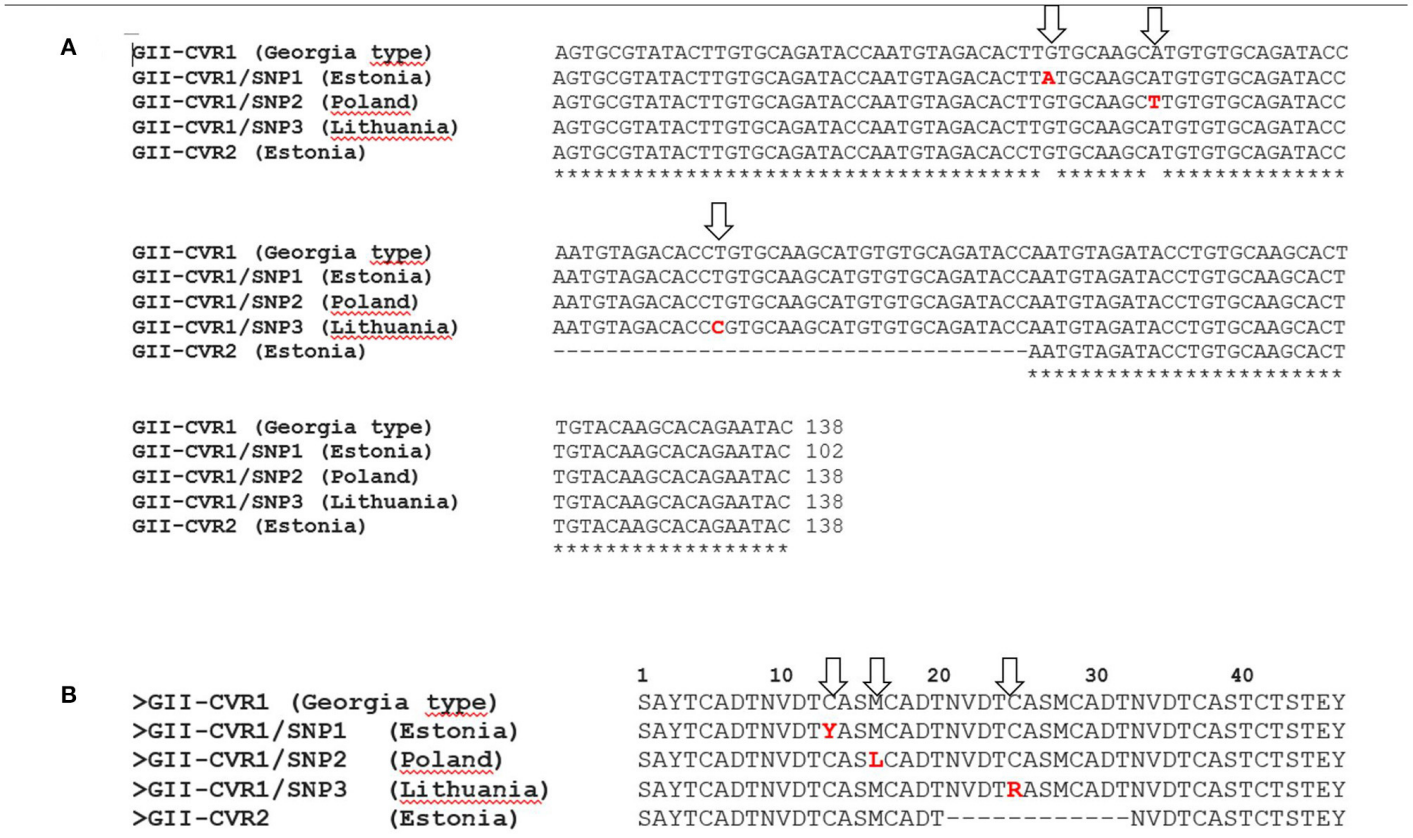
Nucleotide **(A)** and amino acid **(B)** sequence alignment of the variants identified in the central variable region (CVR) of the *B602L* gene in the EU genotype II-ASFVs. In red are showed the variations marked with arrows.

For CVR amplification of the *B602L* gene, 338 samples from ASF cases in wild boar and pig outbreaks collected between 2014 and 2022 in the EU were sequenced. CVR sequences were initially compared with data obtained in previous studies at the EURL of 32 ASFVs (8, 13). Three hundred and seventy (*n* = 370) ASFVs sequenced in this study showed 100% sequence identity to the Georgia 2007/1 strain, thus presenting the CVR region genotype II-variant I (CVR1) with 10 amino acid TRS (BNDBNDBNAA). Sequence alignment revealed the presence in two ASFVs from Poland and Lithuania of two different non-synonymous SNPs resulting in amino acid changes. The identified SNPs differed from the CVR1/SNP1 variant previously described in Estonia (14). Sample Pol17/WB/CASE316 from Poland contained a nonsynonymous (A/T) SNP (CVR1/SNP2) at nucleotide (nt) position 46 of the amplified CVR that is at position 514 of the complete *B602L* protein, resulting in an exchange of methionine (M) for leucine (L) at amino acid position 16 of the CVR (172 of the *B602L* protein). In ASFV from Lithuania (Lt17/WB/Kupiskis/18) a different SNP (CVR1/SNP3) was identified, where thymine (T) was replaced with cytosine (C) at nt position 541 of the complete gene. This transition also resulted in an amino acid change at CVR position 25 that is at position 181 in the complete *B602L* protein, where cysteine (C) was replaced by arginine (R; Figure 1). These variants had not been previously identified in Europe or Asia, as demonstrated by comparison with 495 genotype II-ASFV homologous sequences available in the GenBank.

**Figure 2:**
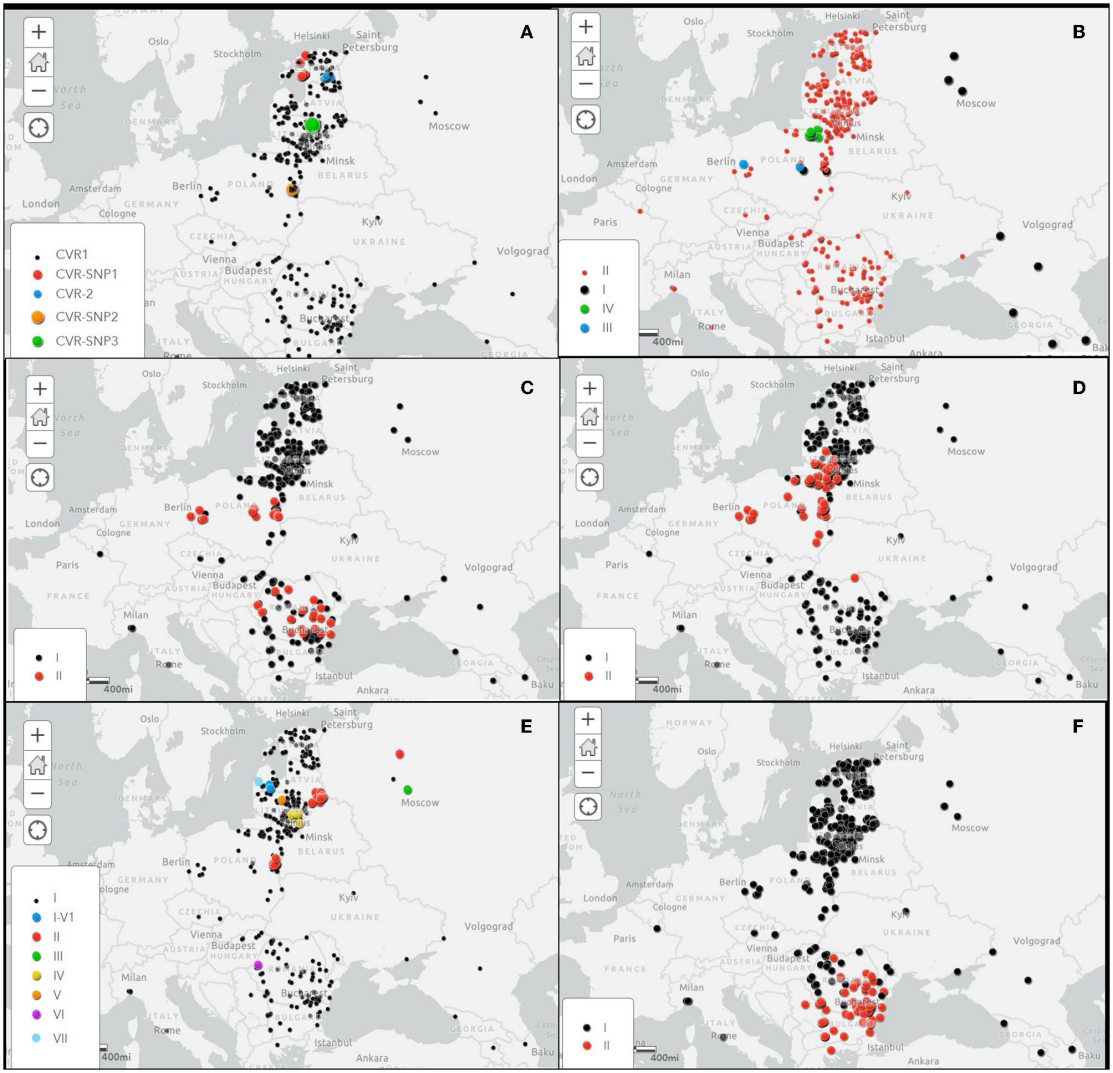
Spatial distribution of investigated gene variants of ASFVs in Europe during the period 2007–2022. **(A)** Central variable region (CVR) within the *B602L* gene, **(B)** Intergenic region (IGR) between *I73R*/*I329L* genes, **(C)** *O174L* gene, **(D)** *K145R* gene, **(E)** IGR between the *9R*/*10R* multigene family 505 (MGF505), and **(F)** ECO2 variants in the IGR between the *I329L*/*I215L* and partial *I215L* gene. Black dots shows the variant 1 100% homologous to the Georgia2007/1 reference strain.

The details of the obtained results are included in the Supplementary Table S1 and the geographical location of the CVR variants identified in this study in Figure 2A.

### 3.2. Analysis of the TRS of the IGR *I73R*/*I329L* (IGR region)

For IGR *I73R*/*I329L* classification, 367 samples from ASF wild boar cases and pigs outbreaks collected between 2012 and 2022 were sequenced within this study. Data were supplemented with IGR sequences of 14 genotype II ASFVs from a previous study (8).

As expected, the IGR-II variant was the most frequent (352/367, 95.91% strains), followed by IGR-IV (10/367 strains, 2.72%), IGR-III (2/367 strains, 0.54%) and IGR-I (strain 2/367, 0.54%). While the IGR-II variant was present in all sampled regions, variants I, III and IV were only detected in Poland (Figure 2A). The IGR-I variant was identified in a sample taken from a wild boar at a distance of about 20 km from the border with Belarus, in Lublin voivodship in March 2017. The same variant was identified in a wild boar hunted in Masovian voivodship in November of the same year, about 160 km from the first detection of the IGR-I variant. The IGR-III variant was linked to an outbreak in domestic pigs in May 2017 in Lublin voivodship, western Poland, and to a wild boar case in Masovian voivodship, east-central Poland in January 2018. The IGR-IV variant was detected in 10 samples collected from wild boar cases in 2018 and 2019 in the eastern Warmian-Masurian voivodship, located in the north-eastern part of Poland, adjacent to Kaliningrad Oblast, Russian Federation. The details of the obtained results are included in the Supplementary Table S1 and the geographical location of the IGR variants in Figure 2B.

In order to improve the picture of ASFV epidemiology in time, the IGR sequences were compared with 540 genotype II ASFV homologous sequences available in the GenBank, resulting in a final data set of 910 IGR sequences. As shown in Table 2, IGR-II was the predominant (92.6%) present in all affected countries of Europe and Asia. The IGR-I variant (frequency 3.8%) has been detected in China, the Russian Federation, Poland, South Korea, and Vietnam, while the IGR-III (frequency 1.6%) in China, South Korea and Vietnam. The IGR-IV variant has only been detected in Poland.

**Table 2:** IGR variants identified in 910 genotype II ASFVs from Europe and Asia.

**Region**	**Country**	**Year**	**No. sequences**		**No. IGR variants**			
			**GenBank**	**This study**	**IGR_I**	**IGR-II**	**IGR-III**	**IGR-IV**
Europe	Georgia	2007–2008	3	–	3	–	–	–
	Armenia	2007	1	–	1	–	–	–
	Azerbaijan	2008	2	–	2	–	–	–
	Russia Federation	2009–2019	38	1	24	14	–	–
	Ukraine	2012- 2016	4	2	–	4	–	–
	Belarus	2013	1	–	–	1	–	–
	Lithuania	2014–2022	102	100	–	102	–	–
	Poland	2014–2020	201	46	2	180	2	17
	Latvia	2014–2021	41	41	–	41	–	–
	Estonia	2014–2022	72	71	–	72	–	–
	Moldova	2016–2018	4	3	–	4	–	–
	Czech Republic	2017–2018	3	2	–	3	–	–
	Romania	2017–2021	46	46	–	46	–	–
	Hungary	2018–2019	5	4	–	5	–	–
	Bulgaria	2018–2020	23	23	–	23	–	–
	Belgium	2018	2	1	–	2	–	–
	Slovakia	2019	1	1	–	1	–	–
	Serbia	2019–2020	14	14	–	14	–	–
	Germany	2020	2	1	–	1	–	–
	Greece	2020	1	1	–	1	–	–
	North Macedonia	2022	6	6	–	6	–	–
	Italy	2022	5	4	–	5	–	–
Asia	China	2018–2020	84	–	1	75	8	–
	Vietnam	2018–2021	174	–	1	170	4	–
	Indonesia	2019–2020	2	–	–	2	–	–
	Mongolia	2019	5	–	–	5	–	–
	Timor-Leste	2019	1	–	–	1	–	–
	South Korea	2019–2020	58	–	1	56	1	–
	India	2020	4	–	–	4	–	–
	Malasya	2021	4	–	–	4	–	–
	Philippines	2021	1	–	–	1	–	–
**Total number**			**910**	**367**	**35**	**843**	**15**	**17**
**Frequency**					**3.8%**	**92.6%**	**1.6%**	**1.9%**

### 3.3. Analysis of the TRS of the *O174L* gene (*O174L* region)

In this study, 382 samples from ASF cases in wild boar and outbreaks in pigs from 2007 to 2022 were used. Most of the samples analyzed (351/382, 91.88%) showed 100% sequence identity with the Georgia strain 2007/1, thus presenting variant I of the *O174L* gene. A 14 nt insertion of CAGTAGTGATTTTT representing variant II of the *O174L* gene (15) was observed in 31 samples (8.11%), including 19 from Romania, 11 from Poland and one from Germany. Consistent with previous studies (15, 16), *O174L* variant II was identified in all samples taken in Lubusz and Masovian voivodeships in central and western Poland, and in four of the six from Lublin, located in the southeast of the country. In contrast, variant II was only identified in one of the 17 samples submitted from Podlaskie, in the northeast of Poland, whilst no variant II samples were observed in the north of Poland. In Romania, *O174L* variant II was detected for the first time in January 2019 in domestic pigs in western Romania, subsequently causing outbreaks in the rest of the country. All viruses tested during this year, except two of wild boar, were grouped within this variant. In contrast, the 2017, 2018 and 2021 Romanian viruses belong to variant I. Except in Germany (27) the *O174L* variant II has not been described in other European and Asian countries as it was determined by comparing 254 homologous sequences available at the GenBank.

The details of these results are included in the Supplementary Table S1 and the geographical location of the *O174L* variants in Figure 2C.

### 3.4. Analysis of the SNP of the *K145R* Gene (*K145R* region)

Conventional sequencing of the *K145R* gene was performed using the same panel of samples as that used for sequencing of the *O174L* gene. The vast majority of the investigated samples (320 of 382, 83.7%) showed 100% identity to the reference strain Georgia 2007/1 representing KP145R variant I. Variant II, characterized by the presence of one SNP (transversion C65167A, referring to Georgia 2007/1) (17) was found in Poland, Lithuania and Romania and in Germany.

In Poland, the *K145R*-II variant was dominant (40/47, 85.1%), while the *K145R*-I variant was identified in only seven ASFVs, two from 2014 index cases and five from wild boar hunted in 2018. It is interesting to note that, except for one hunted wild boar in central Poland, the remaining *K145R*-I variants were collected from areas close to the borders with Belarus, Russian Federation (Kaliningrad Oblast) and Ukraine. In Lithuania, 82 of the 102 ASFVs (80.39%) belonged to variant *K145R*-I and 20 (19.6%) to variant *K145R*-II with strains from Poland and Germany. The first identification of variant II in Lithuania dates from July 2017, when it was detected in domestic pigs on a farm located in Alytus County, on the border with Belarus. Since then, the *K145R*-II variant has been circulating in the wild boar population, mainly in the south of Lithuania, with a sporadic presence in the north of the country. The last cluster of variant II was detected near the border with Kaliningrad Oblast in late 2021-early 2022. Only one of the 42 viruses sequenced from Romania belonged to the *K145R*-II variant group. The sample was collected in 2019 from a wild boar found dead about 6 km of Ukraine border in the Botosani County, in the northern part of Romania. The geographical distribution of the *K145R* variants is showed in Figure 2D.

These sequences were compared to 158 sequences available from GenBank, representing additional genotype II ASFVs obtained from Europe and Asia. The *K145R* variant II was identified in Ukraine in 2016 in Kiev (GenBank Accession No. MN194591) and in 2018 in the Kaliningrad Oblast in seven wild boar ASFVs (GenBank Accession No. OM966714–OM966718, OM966720, OM96672, and OM799941).

### 3.5. Analysis of the TRS of the IGR between the MGF505 *9R* and *10R* genes (MGF region)

**Figure 3:**
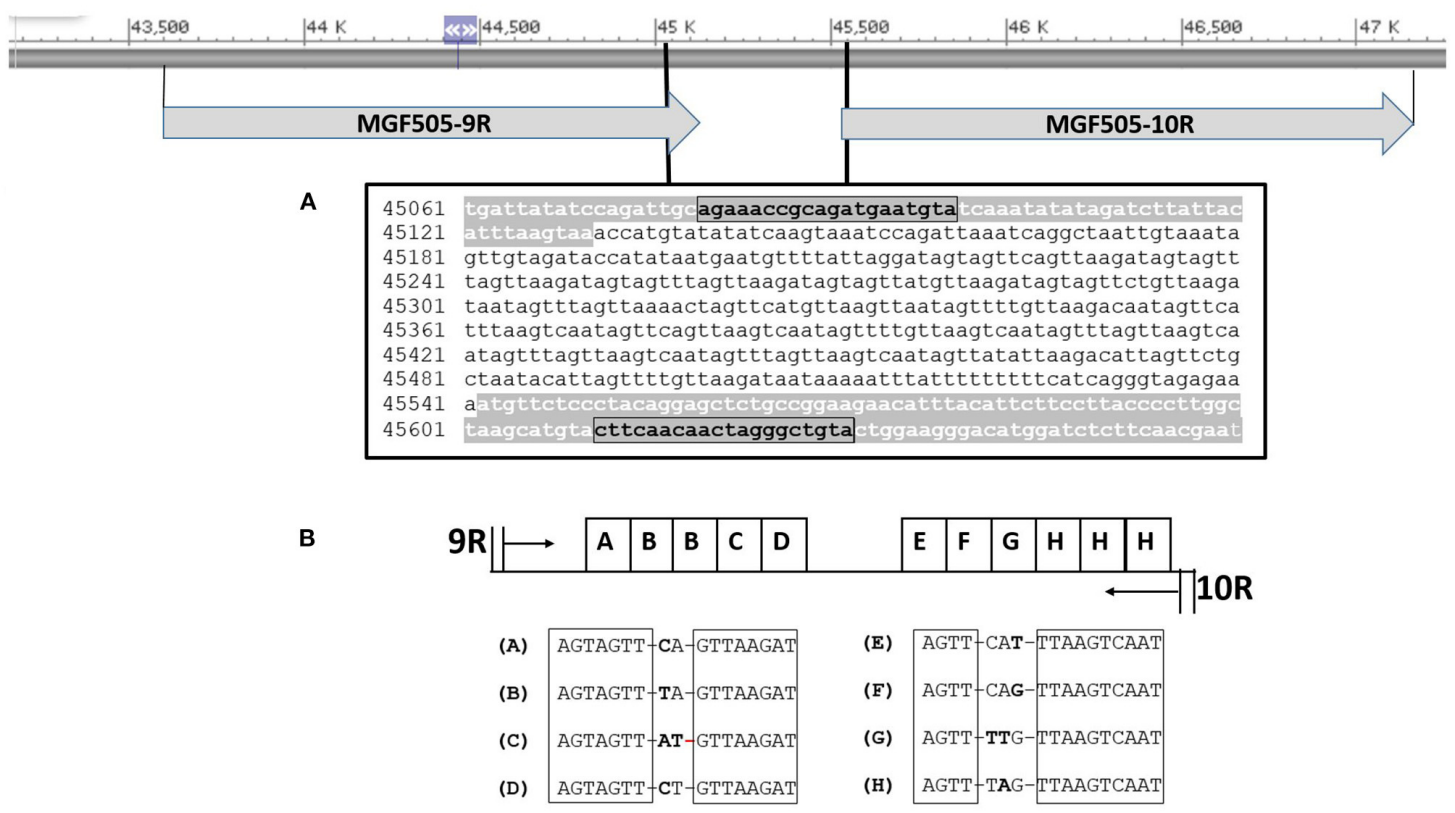
Map showing the location of the IGR between the *9R*-*10R* genes of MGF505 in reference to Georgia ASFV 2007/1. **(A)** Nucleotide sequence of the product amplified with the 505U/505L primers marked in bold. In gray the sequence of the *9R* and *10R* genes is shown. **(B)** Arrangement and identification of intergenic tandem repeat sequences (TRS). The conserved nucleotides in all TRS are boxed.

Amplicons ranging from around 530 to 590 bp were obtained from the 382 genotype II-ASFV isolates sequenced in this study. The molecular basis of this variation involved alterations in the number and type of TRS identified between ORFs *9R*/*10R* (Figure 3B). Two sets of serially repeated DNA sequences could be seen. The first one at residues 45,217–45,302 of the Georgia 2007/1 strain, proximal to the *9R* gene, consisted of five units of 17 nts (AGTAGTTCAGTTAAGAT) with the structure ABBCD. The second set of repeats at residues 45,365–45,467 contains six repetitions of a 17 nts repeat sequence (AGTTCATTTAAGTCAAT) with the structure EFGHHH. The conserved core sequences of the TRS varies in one or two nts (Figure 3B).

The 382 ASFV sequenced were divided into eight different groups based on the number and type of TRS found (Table 3). The largest group, called the MGF-1 group, was 100% homologous to the Georgia 2007/1 reference strain and included 341 of the 382 ASFVs (89.26%) from all sampled countries. The PCR products amplified in this group generated a 551 bp amplicon characterized by the presence of 11 TRS type ABBCD-EFGHHH. The isolates from group 2 (MGF-2) presented a larger amplicon (569 bp) due to the presence of an additional TRS (type ABBBCD-EFGHHH). Twenty-six (6.80%) ASFVs from Russia, Poland, and Latvia were grouped into this group. The MGF-2 variant was initially identified in Russian Federation, in 2012 in a sample taken from an outbreak of domestic pigs. In November 2016, the same variant was found in a wild boar in northeastern Poland, 10 km from the Belarusian border. This was the variant responsible for all but one wild boar cases and domestic pig outbreaks in the same region until June 2017. No further MGF-2 variants has been detected in Poland. In Latvia, the MGF-2 variant was detected for the first time in wild boar in July 2017 in the easternmost region of Latvia that borders Belarus. All wild boar cases that have occurred in this region since then (2017–2021) have been caused by the MGF-2 variant. Interestingly, the only domestic pig outbreak analyzed in this study that occurred in June 2018 in the same region was caused by the MFG-1 variant rather than variant MGF-2. The MGF group's 3, 5, 6, and 7 were represented by a single ASFV from Russia Federation, Lithuania, Romania and Latvia, respectively. The MGF group 4 contained five ASFVs from Lithuania, four from wild boar and one from domestic pig, all of them taken in the eastern counties of Vilna and Utena, border with Belarus. The number of TRS in these groups varied between 10 TRS of groups 4 and 6–13 TRS found in groups 3 and 7 (Table 3).

**Table 3:** Groups based on the analysis of the TRS in the IGR between the *9R*/*10R* genes of the multigene family (MGF) 505. Dashes indicate gaps introduced to enable similarities between sequences to be more easily visualized.

**MGF group**	**Geographical distribution**	**No ASFVs (frequency)**	**Tandem repeat sequences**	**No repeats**	**Amplicon size (bp)**
MGF-1	Armenia, Azerbaijan, Belarus, Belgium, Bulgaria, Czech Republic, Estonia, Georgia, Germany, Greece, Hungary, Italy, Latvia, Lithuania, Moldova, North Macedonia, Poland, Romania, Russia Federation, Serbia, Slovakia, and Ukraine	341/382 (89.26%)	A-BB-CD__EFGHHH	11	551
MGF.2	Latvia, Poland, and Russia Federation	26/382 (6.80%)	A-BBBCD__EFGHHH	12	569
MGF-3	Russia Federation	1/382 (0.26%)	AABBBCD__EFGHHH	13	586
MGF-4	Lithuania	5/382 (1.30%)	A-BB—CD__EFGHH	10	535
MGF-5	Lithuania	1/382 (0.26%)	A-BB—CD__EFGHHHH	12	567
MGF-6	Romania	1/382 (0.26%)	A-B—–CD__EFGHHH	10	535
MGF-7	Latvia	1/382 (0.26%)	A-BBCBCD__EFGHHH	13	586
MGF-8	Latvia	3/382 (3.65%)	A-BB-CD__EFGHHH	11	549

Dashes indicate gaps introduced to enable similarities between sequences to be more easily visualized.

Group 8, with three Latvian ASFVs, showed a three nucleotide deletion at nucleotide position 477 compared to the MGF1 variant (data not shown). This variant, named MGF-1V, was initially detected in November 2017 in a wild boar ASFV in the Brocenu region in the south of the country. Interestingly, the same variant was responsible for the outbreak that occurred on July 10, 2018 in a backyard farm located about 20 km from the initial case.

The details of these results are included in the Supplementary Table S1 and the geographical location of the MGF variants in Figure 2E.

### 3.6. Analysis of the IGR between the *I329L* and *I215L* genes and partial sequencing of the *I215L* gene (ECO2 region)

**Figure 4:**
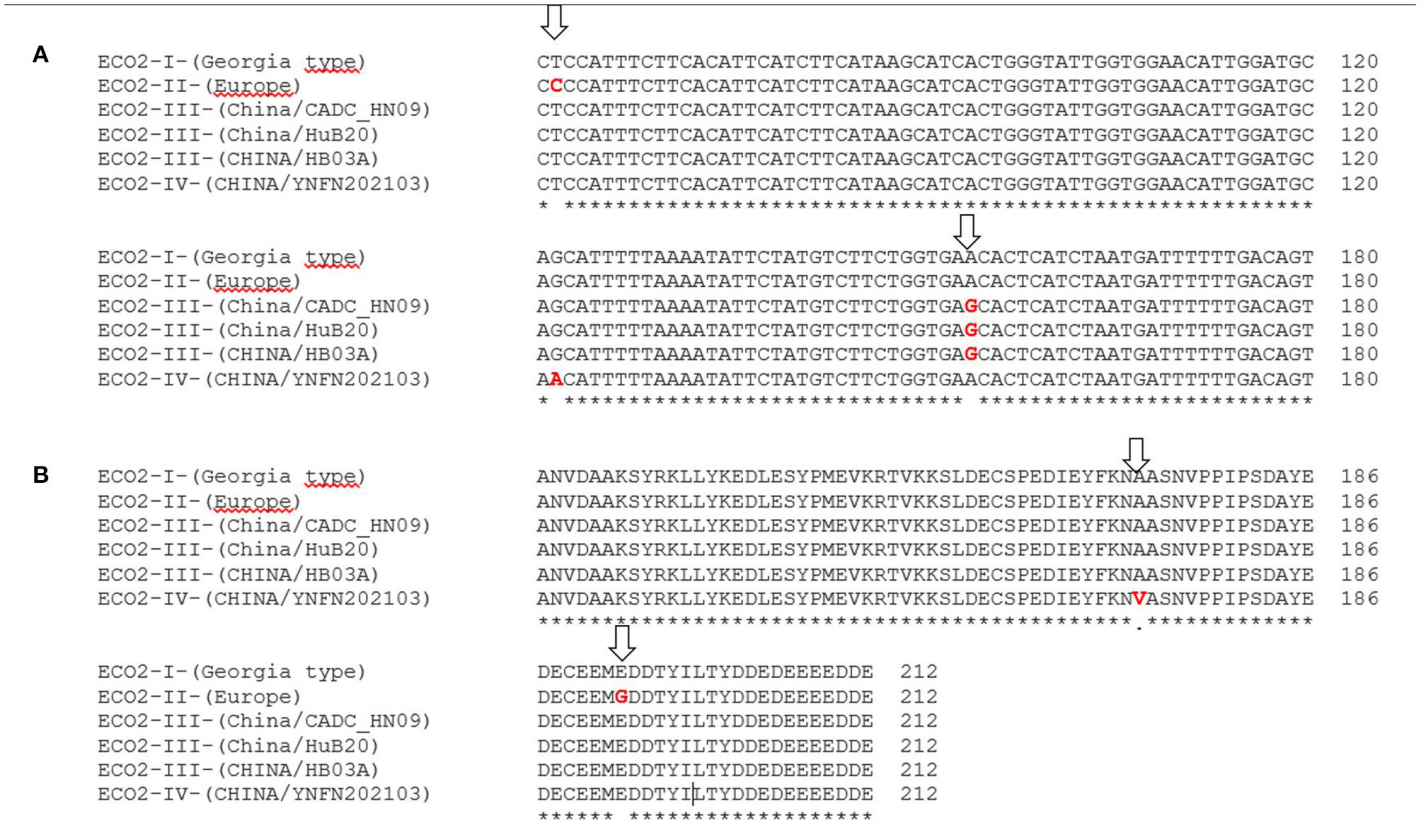
**(A)** Partial nucleotide sequence alignment and **(B)** amino acid alignment of the SNPs identified in the *I215L* gene amplified using the primers ECO2A/2B. In red are showed the variations marked with arrows.

Amplicons of around 600 bp were obtained in the 382 ASFVs that were directly sequenced. Sequences were compared to 60 genotype II ASFVs from Europe and Asia retrieved from the GenBank, giving a final data set of 442 ASFVs. Based on the SNPs found in the *I215L* gene, four variants were identified. The vast majority of ASFVs from Europe and Asia (368/442, 83.25%) were 100% similar to the reference strain Georgia 2007/1 (ECO-I variant). However, in 67/442 (15.1%) of the ASFVs, the presence of a non-synonymous (C/T) SNP (ECO-II variant) was identified at nucleotide position 412 of the amplified fragment, that is at nucleotide 62 of the *I215L* gene (Figure 4A). The SNP resulted in an exchange of glutamic acid (E) for glycine (G) at amino acid position 192 of the *I215L* gene (Figure 4B). The ECO2-II variant was specific to ASFVs from Europe and was initially identified in the ASFVs responsible for the June 2018 domestic pig outbreak in Tulcea County, eastern Romania, on the border with Ukraine. Since then, the ECO2-I and ECO2-II variants circulate simultaneously in Romania, with variant I dominating in the west, while variant 2 predominates in the east, except for a single reported outbreak in Tulcea in 2019 (Supplementary Table S1). All ASFVs from Bulgaria (2018–2020), Serbia (2019–2020), Greece (2020) and North Macedonia (2022) showed a homogeneous nucleotide pattern, with the ECO2-II variant being the only one in circulation. Finally, ECO2-III (SNPA498G) and ECO2-IV (SNPG466A) were identified in two and one ASFVs from China, respectively. The SNP found in the variant 4 resulted in an amino acid exchange of alanine (A) for valine (V) in *I215L* (Figure 4B).

The details of the obtained results are included in the Supplementary Table S1 and the geographical location of the ECO2 variants in Figure 2F.

### 3.7. Genetic group classification of ASFV

Based on the concatenated nucleotide sequences of CVR, IGR *I73R*/*I329L, O174L, K145R*, IGR MGF505*9R*/*10R*, and IGR*I329L*-*I215L*, twenty four genetic groups may be distinguished in Europe (Table 4). The Georgia2007/1 type group, group 1 (CVR-1, IGR-I, *O174L*-I, *K145R*-I, MGF-I, and ECO-I), contains eight non-EU ASFVs (2.1%), collected from 2007 to 2012 in Georgia, Armenia, Azerbaijan, and the Russian Federation. The largest group was group 3 (CVR-1, IGR-II, *O174L*-I, *K145R*-I, MGF-I, and ECO-I) with 192 of the 382 (50.3%) ASFVs collected since 2012 to 2022 and originating from Ukraine, Belarus, Lithuania, Poland, Latvia, Estonia, the Czech Republic, Romania, Moldova, Hungary, Belgium, Slovakia, and the recent isolates from Italy in 2022. The second most frequent cluster was group 19 (CVR-1, IGR-II, *O174L*-I, *K145R*-I, MGF-I, and ECO-II) with 55 ASFVs (14.4%) obtained since 2018 to 2022 from Romania, Bulgaria, Serbia, Greece, and North Macedonia. Group 7 (CVR-1, IGR-II, *O174L*-I, *K145R*-II, MGF-I, and ECO-II) with 29 viruses (7.59%) was identified in ASFVs from Romania, Poland and Lithuania. With the exception of group 6 containing eight ASFVs from Poland and Germany, the remaining clusters were unique to a single country.

**Table 4:** Groups based on the variants identified in the CVR, IGR *I73R*/*I329L* (IGR), *O174L, K145R*, IGR MGF505*9R*/*10R* (MGF), and IGR*I329L*-*I215L* (ECO2) ASFV variable regions.

**Genetic group**	**Geographical distribution (year)**	**No ASFVs (frequency)**	**HOST**	**Genetic variants**					
				**CVR**	**IGR**	***O174L***	***K145R***	**MGF**	**ECO2**
1	Georgia (2007), Armenia (2007, 2008), Azerbaijan (2008), Russia Federation (2009, 2012)	8/382 (2.1%)	EWB, DP	I	I	I	I	I	I
2	Russia Federation (2012)	1/382 (0.26%)	DP	I	I	I	I	II	I
3	Ukraine (2012–2019), Belarus (2013), Lithuania (2014–2020), Poland (2014, 2018), Latvia (2014–2021), Estonia (2014–2022), Czech RP (2017, 2018), Romania (2017–2021), Moldova (2017–2018), Hungary (2018–2019), Slovakia (2019), Italy (2022)	192/382 (50.3%)	EWB, DP	I	II	I	I	I	I
4	Russia Federation (2012)	1/382 (0.26%)	EWB	I	I	I	I	III	I
5	Estonia (2015)	7/382 (1.83%)	EWB	II	II	I	I	I	I
6	Poland (2016, 2019), Germany (2020)	8/382 (2.09%)	EWB, DP	I	II	II	II	I	I
7	Poland (2016–2019), Lithuania (2017–2022), Romania (2019)	29/382 (7.59%)	EWB, DP	I	II	I	II	I	I
8	Poland (2016, 2017)	11/382 (2.88%)	EWB, DP	I	II	I	II	II	I
9	Estonia (2017)	10/382 (2.62%)	EWB, DP	I-SNP1	II	I	I	I	I
10	Poland (2017)	2/382 (0.52%)	EWB	I	I	II	II	I	I
11	Poland (2017)	1/382 (0.26%)	EWB	I-SNP2	II	I	II	I	I
12	Latvia (2017, 2018, 2021)	14/382 (3.66%)	EWB	I	II	I	I	II	I
13	Poland (2017)	1/382 (0.26%)	DP	I	III	II	II	I	I
14	Lithuania (2017)	1/382 (0.26%)	EWB	I-SNP3	II	I	I	I	I
15	Lithuania (2017)	1/382 (0.26%)	EWB	I	II	I	I	V	I
16	Lithuania (2017, 2018)	5/382 (1.31%)	EWB, DP	I	II	I	I	IV	I
17	Latvia (2017, 2018)	3/382 (0.79%)	EWB, DP	I	II	I	I	I-V1	I
18	Poland (2018)	1/382 (0.26%)	EWB	I	III	II	I	I	I
19	Romania (2018, 2021), Bulgaria (2018–2020), Serbia (2019, 2020), Greece (2020), North Macedonia (2022)	55/382 (14.40%)	EWB, DP	I	II	I	I	I	II
20	Poland (2018, 2019)	10/382 (2.62%)	EWB	I	IV	I	II	I	I
21	Romania (2019)	7/382 (1.83%)	EWB, DP	I	II	II	I	I	I
22	Romania (2019)	12/382 (3.14%)	EWB, DP	I	II	II	I	I	II
23	Lithuania (2020)	1/382 (0.26%)	EWB	I	II	I	I	VII	I
24	Romania (2021)	1/382 (0.26%)	DP	I	II	I	I	VI	I

EWB, European wild boar; DP, domestic pig.

Per country, the greatest variability was found in the ASFVs from Poland distributed in nine different groups (3, 6, 7, 8, 10, 11, 13, 18, and 20), followed by six groups from Romania (3, 7, 19, 21, 22, and 24) and five groups from Lithuania (3, 7, 14, 15, and 16). In Estonia and Latvia 3 and 4 groups have been respectively identified, with group 3 being common to both countries, while groups 5, 9 (Estonia) and 12, 17, and 23 (Latvia) were exclusive to each country. Except in the Russian Federation with three identified groups (1, 2, and 4), in the remaining countries, circulating ASFVs were grouped into a single group. Regarding the host, most of the groups (11/24) included viruses obtained from both domestic pigs and wild boar (11/24), 10 were specific to wild boar and only three from domestic pigs.

The details of the obtained results are included in the Supplementary Table S1 and Table 4.

## 4. Discussion

One of the main gaps in the epidemiology of the ASFV is to differentiate the origin of the outbreaks or to trace the dynamics of the infection in the affected populations once a certain genotype is circulating in a country or region. This is mainly due to the large size of the ASFV genome that ranges from 170 to 193 kbp, which makes sequencing the entire genome difficult, and its low mutation rate as it is a DNA virus (2). In this study, we have investigated six variable regions of the ASFV genome and examined their use in distinguishing between closely related genotype II ASFVs circulating in Europe since 2007 up to 2022. Two approaches were utilized to study genome variability. First, the sequencing of four previously described regions of the genome that contain TRS or SNPs; the CVR (13), the IGR between the *I73R*-*I329L* genes (8), the *O174L* gene (15) and the partial sequencing of the *K145R* gene (16). Second, we designed two new PCRs to amplify two additional ASFV variable regions characterized by the presence of TRS located in the IGR between the *9R*−*10R* genes of MGF 505 (20, 21) and in the IGR of the *I329L*–*I215L* genes (22). The aim was to obtain additional markers capable of differentiating between the viruses circulating in Europe, especially those associated with the recent outbreaks that occurred in Italy and North Macedonia in 2022.

The CVR region is frequently the subject of sequence analysis due to single mutations capable of resolving phylogenies at the regional level (13, 28–34). Within genotype II-ASFVs, and despite the large number of outbreaks in Europe and Asia, CVR variants have only been described in Estonia, classifying the isolates into three different groups; CVR1 (Georgia2007/1 type), CVR2 characterized by an amino acid deletion of CASMCADTNVDT, and CVR1-SNP1 having a nucleotide mutation (14). Even though the resolving power of this gene region in isolates from Africa (28–34) and the polymorphisms observed in isolates from Estonia (14), only two SNP-based CVR variants were detected in this study in wild boar viruses from Poland (CVR1-SNP2) and Lithuania (CVR1-SNP3). Recent studies describe the presence of additional CVR variants in isolates from Russia (35) and China (19) based on the presence of SNPs. However, the variants described in this study were unique and were not further identified in any of the ASFVs sequenced or available in the GenBank.

The IGR *I73R*-*I329L* is characterized by the presence of 10-nucleotide tandem repeats of “TATATAGGAA” (8) that allow genotype II-ASFVs to be classified into one of the four variants identified so far; IGR-I (two copies), IGR-II (three copies), IGR-III (four copies) and IGR-IV (five copies) (18). According to the results of this study, the IGR-II variant, which emerged in 2012 or even earlier in the Russian Federation (8, 36), is the most frequently identified in the EU-ASFVs. This variant was responsible for the first cases in 2014 in Lithuania and Poland on the border with Belarus and has spread widely throughout all affected regions (8). The four IGR variants were only detected in Poland, being the first description in the EU for the IGR-I and IGR-III variants. Outside the EU these variants were circulating in China, Russia Federation, South Korea and Vietnam (17–19, 37–39). Consistent with what was described by Mazur-Panasiuk et al. (16), IGR-IV was exclusively identified in northern Poland on the border with Kaliningrad Oblast, Russia.

Previous sequencing studies of the *O174L* gene classified the isolates from Poland into two different variants, with variant II being the predominant one in the western part of the country and responsible for the ASF outbreaks in Germany (15, 16, 27). In our study, sequencing of the *O174L* gene revealed the presence of the *O174L*-II variant in Poland, as expected, but also in Romania, both in wild boar and domestic pigs. Similarly, the *K145R* variant II, described in Poland, Germany and Russia Federation (Kaliningrad Oblast) (15, 16), was found in Poland, Lithuania, and Romania.

The results obtained after the analysis of the four published variable regions did not allow to identify the origin of the outbreaks that occurred in 2022 in Italy and North Macedonia. For this, the IGR between MGF 505 *9R* and *10R* (MGF 505 *9R*/*10R*) characterized by the presence of TRS (20, 21) was sequenced. Eight MGF variants were identified based on the number and type of TRS. The majority (about 89%) of the ASFVs were 100% homologous to the Georgia 2007/1 strain and were classified as the MGF-1 variant, including those from the 2022 outbreaks. The MGF-2 variant, originating from the Russia Federation in 2012, formed a heterogeneous group with viruses from Poland and Latvia collected near the Belarusian border. Groups 3 to 8 were country specific. Despite its low utility in determining the origin of ASF 2022 epidemics, the IGR between the MGF*9R*/*10R* genes showed higher resolving power than the CVR, IGR, *O174L*, and *K145R* regions. This fact gives this region great potential as a molecular marker, especially in endemic regions where ASF has established within the wild boar population. To identify the origin of the 2022 outbreaks, a new PCR was designed that amplifies the IGR between the *I329L* and *I215L* genes and the carboxy terminus of the *I215L* gene (22). Partial sequencing of the *I215L* gene identified a SNP within the *I215L* gene, differentiating two geographically distinct genetic variants circulating in Europe. The ECO2-II variant, characterized by the presence of the SNP and initially identified in Romania in 2018, was responsible for the 2022 outbreaks in North Macedonia. This variant is circulating in Romania, Bulgaria, Serbia, Greece, and North Macedonia, forming a geographically distinct genetic group in Europe.

The combined analysis of the results obtained in the six ASFV genome regions sequenced enabled 382 ASFV isolates from Europe to be divided into 24 genetic groups. Group 1 is the oldest containing the isolate from Georgia in 2007 and is classified as the reference group, and includes historical genotype II-ASFVs from Europe. Group 3, the largest with 50.3% of ASFV isolates, includes the IGR-II variant identified in the EU “index” cases in 2014 in the vicinity of the Belarusian border. This group comprises ASFV from 12 countries, nine from the EU, including recent ASFV isolated in Italy in 2022. The second largest group (14.4%), group 19, contains the IGR II and ECO2-II variants and comprises viruses from Southeast Europe. This group was initially identified in 2018 in an outbreak on a backyard farm located in Tulcea, southeastern Romania, on the border with Odessa, Ukraine. Regarding the fact that this variant was completely absent in Romania, where group 3 was unique, it could be assumed that the disease probably jumped to Tulcea from neighboring countries such as Ukraine, but its exact origin cannot be determined based on the molecular data available from Ukraine. It was not further identified in Romania until January 2021, spreading throughout the country. This genetic group was subsequently identified in Bulgaria in August 2018, in Serbia in 2019, in Greece in 2020 and in North Macedonia in 2022. In these countries it is the only group identified.

In contrast, the ASFVs from Romania were grouped into 6 distinct groups (3, 7, 19, 21, 23, 24). Interestingly, the 2019 epidemic wave, despite taking place in a short space of time (40), was caused by two distinct genetic groups specific only to Romania. Group 21, containing the IGR-II and *O174L*-II variants, was initially detected in January 2019 in the western regions and had a wide geographic spread, being identified in the north and east. Group 22 with the IGRII, *O174L*-II and ECO2-II variants formed a differentiated cluster in the southeast of the country. A similar unexpected introduction was observed in 2021 on a backyard farm in Arad, in the westernmost part of Romania, caused by a new genetic group, group 24 possessing the MGF-6 variant, never before identified. The origin of these genetic groups is unknown mainly due to the lack of information from neighboring non-EU countries. The epidemiological analysis carried out by Andraud et al. (40) clearly identified human activity and the distribution of the pig population as the main risk factors for the spread of ASF in epidemic waves in Romania. However, from the molecular data obtained in this study, we cannot exclude the role of wild boars in the infection of domestic pigs, probably due to the low level of biosecurity in backyard farms, predominant in the country (40). Although the situation in Romania cannot be directly translated to intensive pig farming countries, the results of this study highlight the need for strict biosecurity measures on farms and during transport to prevent the transmission of ASF on a large scale. Cluster 7 (IGR-II and *K145R*-II), also circulating in Romania, was associated with a wild boar case that occurred in the north of Botosani County, about 2 km from the Ukrainian border, assumed to have been introduced through the wild boar migration. This group, described by Mazur-Panasiuk et al. (16) as the most typical for Poland since 2016, was also identified in Lithuania in 2017 in an outbreak of domestic pigs on the Ukrainian border in Alytus County, southern Lithuania. Subsequently, two temporal a spacial differentiated groups were formed; the 2017–2018 group originating from Alytus that spread to central and northern Lithuania, and the 2021–2022 group identified in Taurage County, on the border with Kaliningrad Oblast. This same genetic group was identified in a wild boar hunted in the Kaliningrad Oblast in 2018 (ASFV/Kaliningrad_18/WB-12516; GenBank OM966720.1), so reaffirming that wild boar is playing a key role in the reintroduction of the ASFVs in affected countries. Three additional groups, 14 (IGRII and CVR-SNP1), 15 (IGR-II and MGF-5), and 16 (IGR-II, MGF-4) were sporadically detected in Lithuania in 2017 and 2018. It should be noted that group 17, detected on the border with Belarus, made it possible to genetically link isolates from domestic pigs and wild boars in Utena County. Indeed, 11 of the 24 groups clustered both wild boar and domestic pigs ASFVs.

The higher variability found in isolates from Poland with nine of 24 groups identified could be explained by the long persistence of the disease since 2014 within the wild boar population. However, in Lithuania, Estonia, and Latvia, where the disease has also been endemic since 2014, group 3 detected in the initial outbreaks was clearly the predominant one, with additional spatially and geographically restricted groups. Forth et al. (27) has recently described an increased mutation rate in the affected region of Germany, where the disease was introduced in 2020. Twenty-two ASFV isolates from Germany diverged into five clearly distinguishable lineages with at least 10 different variants characterized by high-impact mutations. Notably, all of the new variants share a 3′-end frameshift mutation of the PolX DNA polymerase *O174L* gene, initially described in Poland by Mazur-Panasiuk et al. (16), suggesting a causal role as a possible mutator gene. This could explain the greater variability found in countries where the *O174L*-II variant circulates, such as Poland and Romania, compared to countries where variant I is predominant.

Our results confirmed that genomic regions containing TRS generally occur in regions where the disease has long persisted within the wild boar population, appearing sporadically in domestic pigs. These regions are of particular interest in terms of standard genotyping procedures because of the difference in the length of the PCR product, which is convenient to observe during regular agarose electrophoresis. In addition to tandem repeats, SNPs represent an attractive molecular tool that allows discrimination of closely related ASFV genotype II strains into clearly distinct groups obtained for different space and time. Whole genome sequencing, while essential to identify new genetic markers, is not a feasible routine genotyping method due to the complexity of the required sequence analysis vs. the final results, the time consuming, high cost, and specialized personnel required. In this study, we describe a new multi-gene approach sequencing method that distinguishes European II-ASFV genotypes into 24 distinct groups by sequencing six independent ASFV genomic regions, including the never-before-described IGR 1329L–*I215L*, named ECO2 region. The introduction of a subtyping method into routine diagnosis within affected areas worldwide may help to identify potential origins of the disease and provide a deeper understanding of the spatial and temporal trajectories and routes of the disease. Additional sequencing of other genetic markers could cluster genotype II ASFV isolates at higher resolution and cannot be ruled out.

## Data availability statement

The original contributions presented in the study are included in the article/Supplementary material, further inquiries can be directed to the corresponding author.

## Author contributions

CG was the principal investigator, analyzed the sequences, wrote the manuscript, and designed the experiments. NC performed sequencing analysis. ASo and RN tested the clinical samples for ASF diagnostic processing the samples and did the PCR. ID and LK provided the clinical samples from North Macedonia and Latvia. EM purified the sequences and did the SANGER sequencing. CP and ASi did the conventional PCR for sequencing. EI, DD, VM, EC, IN, MF, FF, PV, and SP provided the clinical samples from affected ASF areas of Bulgaria, Romania, Serbia, Greece, Estonia, Poland, Italy, the Czech Republic, and Lithuania. AM is the EURL Director and overseeing all experimental work. All authors have read and approved the manuscript.
